# *Arabidopsis thaliana* CYCLIC NUCLEOTIDE‐GATED CHANNEL2 mediates extracellular ATP signal transduction in root epidermis

**DOI:** 10.1111/nph.17987

**Published:** 2022-02-20

**Authors:** Limin Wang, Youzheng Ning, Jian Sun, Katie A. Wilkins, Elsa Matthus, Rose E. McNelly, Adeeba Dark, Lourdes Rubio, Wolfgang Moeder, Keiko Yoshioka, Anne‐Aliénor Véry, Gary Stacey, Nathalie Leblanc‐Fournier, Valérie Legué, Bruno Moulia, Julia M. Davies

**Affiliations:** ^1^ Department of Plant Sciences University of Cambridge Cambridge CB2 3EA UK; ^2^ Institute of Integrative Plant Biology School of Life Science Jiangsu Normal University Xuzhou 221116 China; ^3^ Facultad de Ciencias Departamento de Botánica y Fisiología Vegetal Universidad de Málaga Málaga 29071 Spain; ^4^ Department of Cell & Systems Biology University of Toronto Toronto ON M5S 3B2 Canada; ^5^ Biochimie & Physiologie Moléculaire des Plantes UMR Université Montpellier CNRS INRAE Institut Agro Montpellier 34060 France; ^6^ 14716 Divisions of Plant Science and Technology University of Missouri Columbia MO 65211 USA; ^7^ Université Clermont Auvergne INRA PIAF Clermont‐Ferrand F‐63000 France

**Keywords:** *Arabidopsis*, calcium, CNGC4, CYCLIC NUCLEOTIDE‐GATED CHANNEL2 (CNGC2), depolarization, extracellular ATP, plasma membrane potential, root epidermis

## Abstract

- Damage can be signalled by extracellular ATP (eATP) using plasma membrane (PM) receptors to effect cytosolic free calcium ion ([Ca^2+^]_cyt_) increase as a second messenger. The downstream PM Ca^2+^ channels remain enigmatic. Here, the *Arabidopsis thaliana* Ca^2+^ channel subunit CYCLIC NUCLEOTIDE‐GATED CHANNEL2 (CNGC2) was identified as a critical component linking eATP receptors to downstream [Ca^2+^]_cyt_ signalling in roots.
- Extracellular ATP‐induced changes in single epidermal cell PM voltage and conductance were measured electrophysiologically, changes in root [Ca^2+^]_cyt_ were measured with aequorin, and root transcriptional changes were determined by quantitative real‐time PCR. Two *cngc2* loss‐of‐function mutants were used: *cngc2‐3* and *defence not death1* (which expresses cytosolic aequorin).
- Extracellular ATP‐induced transient depolarization of Arabidopsis root elongation zone epidermal PM voltage was Ca^2+^ dependent, requiring CNGC2 but not CNGC4 (its channel co‐subunit in immunity signalling). Activation of PM Ca^2+^ influx currents also required CNGC2. The eATP‐induced [Ca^2+^]_cyt_ increase and transcriptional response in *cngc2* roots were significantly impaired.
- CYCLIC NUCLEOTIDE‐GATED CHANNEL2 is required for eATP‐induced epidermal Ca^2+^ influx, causing depolarization leading to [Ca^2+^]_cyt_ increase and damage‐related transcriptional response.

## Introduction

Extracellular ATP (eATP) has been shown to contribute to plant growth and development, stress responses, immunity, and damage (Matthus *et al*., 2019a). Two plasma membrane (PM) coreceptors for eATP, DOES NOT RESPOND TO NUCLEOTIDES1 (P2K1/DORN1) and P2K2, have been identified recently in *Arabidopsis thaliana*, with P2K1/DORN1 transphosphorylating P2K2 (Choi *et al*., 2014; Pham *et al*., 2020). P2K1/DORN1 commands an eATP‐dependent transient increase of cytosolic free calcium ions ([Ca^2+^]_cyt_) as a second messenger (Choi *et al*., 2014). The root [Ca^2+^]_cyt_ response to eATP (the ‘signature’) has a greater reliance on Ca^2+^ influx across the PM than the release of Ca^2+^ from intracellular stores (Demidchik *et al*., 2009; Rincón‐Zachary *et al*., 2010). Lowering external Ca^2+^ from 10 to 0.1 mM causes an 85% decrease in the [Ca^2+^]_cyt_ response (Demidchik *et al*., 2003). Ca^2+^ influx across the PM helps explain the depolarizing effect that eATP has on root PM voltage (Lew & Dearnaley, 2000; Dindas *et al*., 2018), especially given that eATP causes instantaneous [Ca^2+^]_cyt_ increase and a cytosolic acidification consistent with PM H^+^‐ATPase inhibition in Arabidopsis roots (Waadt *et al*., 2020). Indeed, patch clamp electrophysiology has revealed eATP and P2K1/DORN1‐dependent Ca^2+^‐permeable channel conductances in Arabidopsis root epidermal PM (Demidchik *et al*., 2009; Wang *et al*., 2018, 2019) that could contribute to PM depolarization and [Ca^2+^]_cyt_ increase. However, the identity of the channels remains unknown. Here, data support the involvement of a CYCLIC NUCLEOTIDE‐GATED CHANNEL (CNGC).

Arabidopsis has a family of 20 CNGC subunits, with members contributing to [Ca^2+^]_cyt_ signatures evoked by abiotic stress, pathogen attack, and hormones (Jarratt‐Barnham *et al*., 2021). Because eATP accumulates during pathogen infection and acts as a damage‐associated molecular pattern (DAMP) that drives a transcriptional response through P2K1/DORN1 (Choi *et al*., 2014; Jewell *et al*., 2019; Kumar *et al*., 2020), CNGCs involved in pathogen sensing could also be acting in the eATP pathway. CYCLIC NUCLEOTIDE‐GATED CHANNEL2 is a key candidate for testing, as it operates in root signalling (Chakraborty *et al*., 2021), it is involved in both DAMP and pathogen‐associated molecular pattern (PAMP) signalling, and it generates a PM hyperpolarization‐activated Ca^2+^‐permeable channel conductance (Qi *et al*., 2010; Tian *et al*., 2019). Cyclic Nucleotide‐Gated Channel2's closest paralogue, CNGC4, can interact with CNGC2, and these two subunits are hypothesized to form a heteromeric channel in PAMP signalling (Chin *et al*., 2013; Tian *et al*., 2019). Cyclic Nucleotide‐Gated Channel2 and CNGC4 could potentially work together in the eATP pathway.

Here, two Arabidopsis *cngc2* loss of function mutants were used: *cngc2‐3* and *defence not death1* (*dnd1*; which expresses cytosolic aequorin). Extracellular ATP‐induced depolarization of PM voltage has been used as a diagnostic of PM Ca^2+^ channel activity in single epidermal and cortical root cells. Results show an absolute requirement for CNGC2 but not CNGC4 in the epidermis. Patch clamp electrophysiological analysis of eATP‐induced PM Ca^2+^ influx conductance of epidermal cells confirmed an absolute requirement for CNGC2. Both root eATP‐induced [Ca^2+^]_cyt_ signature and transcriptional response were impaired by loss of CNGC2 function.

## Materials and Methods

### Plant material

Arabidopsis lines were in the Columbia (Col‐0) ecotype. *dorn1‐1*, *dorn1‐3*, *p2k2*, and *p2k1p2k2* mutants were as described previously (Choi *et al*., 2014; Pham *et al*., 2020). *cngc2‐3* (transfer DNA (T‐DNA) insertion line Salk‐066908) was described previously by Chin *et al*., 2013. Complemented *cngc2‐3* was generated with the CNGC2 coding sequence under the control of its endogenous promoter (Supporting Information Methods S1). *dnd1 cngc2* loss‐of‐function mutant constitutively expressing cytosolic (apo)aequorin was described by Qi *et al*., 2010. *cngc4‐5* (SALK_081369; Tian *et al*., 2019) was obtained from the Nottingham Arabidopsis Stock Centre. Genotyping of insertional and complemented mutants is described in Methods S1. Primers are listed in Table S1. Growth conditions are described in Methods S2. Plants at 7–14 d old were used unless stated otherwise.

### Membrane potential measurements

Plasma membrane potential *E*_m_ of root elongation zone cells was measured using a glass microelectrode. A plant was fixed in a plexiglass chamber and immersed in assay solution (10 ml) containing 2 mM calcium chloride (CaCl_2_; with or without 5 mM ethylene glycol‐bis(β‐aminoethyl ether)‐*N*,*N*,*N*′,*N*′‐tetraacetic acid) (EGTA) or with or without 0.5 mM lanthanum chloride (LaCl_3_)), 0.1 mM potassium chloride (KCl), 1 mM MES–Tris (pH 6.0) for at least 30 min before impalement. Microelectrode construction, recording circuitry, and impalement are described in Methods S3. After observing a stable *E*_m_ (> 6 min), eATP (ATP magnesium salt (MgATP) or ATP disodium salt (Na_2_ATP); Sigma) was added to the chamber (final concentration 300 µM in the assay medium, pH 6.0). In controls, magnesium sulphate (MgSO_4_) or sodium sulphate (Na_2_SO_4_) was added.

### Patch clamp recordings

Protoplasts were isolated from root elongation zone epidermis, with origin confirmed using the N9093 epidermal‐specific green fluorescent protein reporter line as described by Wang *et al*. (2019). Details of isolation, patch clamp solutions, and protocols are in Methods S4.

### Cytosolic free calcium ion measurement

Excised primary roots of Col‐0 and *dnd1* expressing cytosolic (apo)aequorin were used for luminescence‐based quantification of [Ca^2+^]_cyt_. Roots were placed individually into a 96‐well plate (one root per well) and incubated overnight at room temperature in darkness with 10 µM coelenterazine in 100 µl of buffer: 2 mM CaCl_2_, 0.1 mM KCl, 1 mM MES–Tris (pH 5.6). CaCl_2_ was included to maintain a similar level to that of the growth medium. Samples were washed with coelenterazine‐free buffer and left to recover for at least 20 min in darkness. A FLUOstar Optima plate reader (BMG Labtech, Ortenberg, Germany) was used to record luminescence as described in Matthus *et al*. (2019b). [Ca^2+^]_cyt_ was calculated as described by Knight *et al*., 1997.

### Analysis of gene expression

Total RNA was extracted from roots (frozen in liquid nitrogen) using the RNAeasy Plant Mini Kit (Qiagen) and subjected to DNase I treatment (RNAse‐free DNAse kit; Qiagen). Complementary DNA (cDNA) was synthesized using the QuantiTect Reverse Transcription Kit (Qiagen). Quantitative real‐time (qRT)‐PCR was performed in a Rotor‐Gene 3000 thermocycler with the Rotor‐Gene™ SYBR^®^ Green PCR Kit (Qiagen). *UBQ10* and *TUB4* acted as internal controls. Primers are listed in Table S1. Further details are in Methods S5.

### Statistical analysis

Data normality was first analysed with the Shapiro–Wilk test in R. Student's *t*‐test or Tukey's honestly significant difference was used for parametric data comparison, whereas the Mann–Whitney *U* test was used to compare the nonparametric data.

## Results

### AtCNGC2 mediates the extracellular‐ATP‐induced depolarization of root epidermal plasma membrane voltage and does not require AtCNGC4

**Fig. 1:**
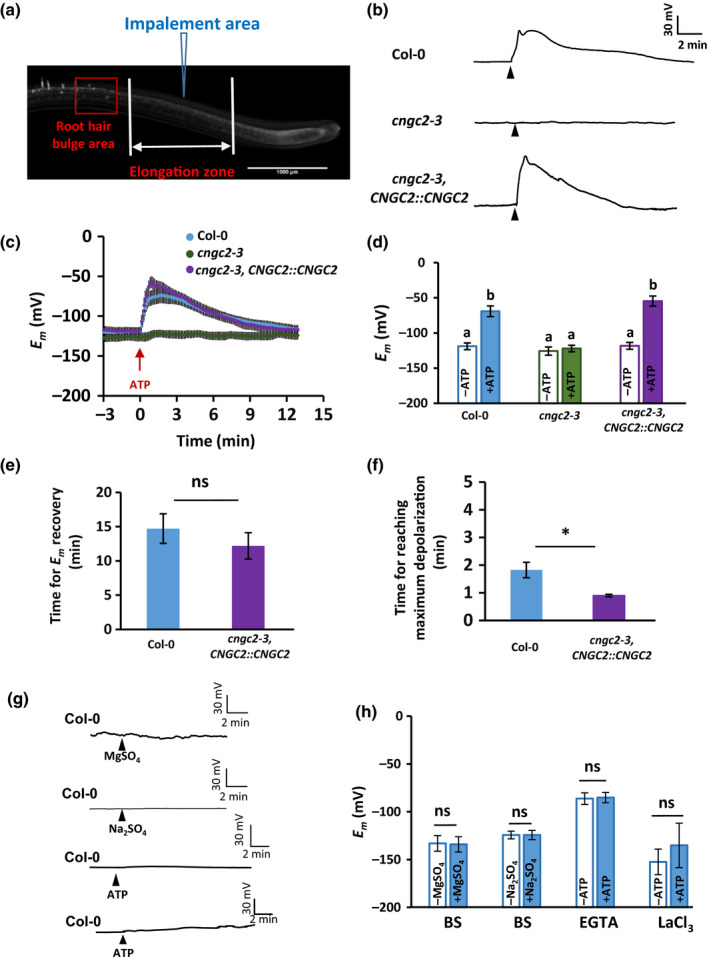
CYCLIC NUCLEOTIDE‐GATED CHANNEL2 (CNGC2) is required for extracellular ATP (eATP)‐induced depolarization of primary root elongation zone epidermal plasma membrane potential *E*_m_. (a) A representative example of an *Arabidopsis* Col‐0 root indicating the elongation zone where a single epidermal or cortical cell was impaled with a microelectrode (represented by the blue triangle). Bar, 0.1 cm. (b) Representative epidermal *E*_m_ recordings from Col‐0, *cngc2‐3*, and *cngc2‐3,CNGC2::CNGC2* treated with 300 µM eATP (black triangles indicate addition). (c) Mean ± SE time courses of the response to 300 µM eATP for Col‐0 (*n* = 9), *cngc2‐3* (*n* = 9), and *cngc2‐3,CNGC2::CNGC2* (*n* = 5). The chemical addition time was set to zero on the *x*‐axis. (d) Comparison of mean ± SE *E*_m_ before eATP (−ATP) and after eATP treatment (+ATP; maximum depolarization). Different lower‐case letters on the top of vertical bars indicate significant difference between means (*P* < 0.05). (e) Col‐0 and *cngc2‐3,CNGC2::CNGC2* took similar times for *E*_m_ to recover from depolarization. (f) *cngc2‐3,CNGC2::CNGC2 E*_m_ depolarized more rapidly than Col‐0 in response to eATP. (g) Representative epidermal *E*_m_ recordings from Col‐0. Top trace: response to 300 µM magnesium sulphate (MgSO_4_; *n* = 8); second trace: response to 300 µM sodium sulphate (Na_2_SO_4_; *n* = 6); third trace: response to 300 µM eATP in the presence of 5 mM ethylene glycol‐bis(β‐aminoethyl ether)‐*N*,*N*,*N*′,*N*′‐tetraacetic acid) (EGTA; *n* = 5); bottom trace: response to 300 µM eATP in the presence of 0.5 mM lanthanum chloride (LaCl_3_; *n* = 3). Mean time courses are in Supporting Information Fig. S1. (h) Comparison of mean ± SE *E*_m_ before and after addition of 300 µM MgSO_4_ or Na_2_SO_4_ to bath solution (BS) and before and after addition of eATP in the presence of 5 mM EGTA or 0.5 mM LaCl_3_ in the BS. Bath solution contained 2 mM calcium chloride, 0.1 mM potassium chloride, 1 mM MES–Tris (pH 6.0). *, *P* < 0.05; ns, not significant.

The stable resting membrane voltage *E*_m_ of a single Col‐0 root elongation zone epidermal cell (Fig. 1a) was significantly but transiently depolarized by 300 µM eATP (Fig. 1b). This concentration of eATP was found previously to activate a PM Ca^2+^ influx conductance in this cell type (Wang *et al*., 2019). Mean maximal depolarization from −118.9 ± 4.8 to −69.2 ± 7.6 mV (Fig. 1c,d; Table S2) occurred 1.8 ± 0.3 min after eATP application (MgATP or Na_2_ATP), and *E*_m_ recovered fully after 14.7 ± 2.2 min (Fig. 1e,f) in the continued presence of eATP. In controls, neither 300 µM MgSO_4_ nor 300 µM Na_2_SO_4_ (Figs 1g,h, S1a,b) affected *E*_m_, confirming that the response was due to eATP. Incubation with 5 mM EGTA (to chelate extracellular Ca^2+^) abolished the response to 300 µM eATP (Figs 1g,h, S1c), showing that depolarization required Ca^2+^ influx. However, as EGTA treatment resulted in a less negative *E*_m_ that could have compromised depolarization, a further test of Ca^2+^ influx was conducted. Addition of 0.1 mM LaCl_3_ as a blocker of PM Ca^2+^‐permeable channels prevented significant depolarization by eATP (Figs 1g,h, S1d). The loss‐of‐function *cngc2‐3* mutant (T‐DNA insert in second exon) and the complemented *cngc2‐3,CNGC2::CNGC2* mutant (Fig. S2a–c) were then analysed. Expression levels of *P2K1/DORN1* and the coreceptor *P2K2* were normal in *cngc2‐3* roots, indicating that eATP perception itself would be unimpaired (Fig. S2d). There were no significant differences in resting *E*_m_ between genotypes (Table S2). In contrast to Col‐0, 300 µM eATP failed to depolarize *cngc2‐3 E*_m_ (Fig. 1b–d; Table S2). Complementation fully restored the mutant's *E*_m_ response to eATP (depolarization and recovery time) (Fig. 1b–e), but maximum *E*_m_ depolarization occurred sooner than in Col‐0 (Fig. 1f). This may reflect the approximately doubled abundance of *CNGC2* transcript in the complemented mutant, although this was not statistically significant (Fig. S2e). To verify the *cngc2‐3* results, the *CNGC2 dnd1* mutant (Fig. S3a–c) was also tested. This has a single point mutation causing a stop codon in the third exon and expresses cytosolic aequorin (Qi *et al*., 2010). Resting *dnd1 E*_m_ was not significantly different to those of other genotypes and was unaffected by eATP treatment (Fig. S3d–f; Table S2). These results show that the eATP‐induced and Ca^2+^‐dependent PM *E*_m_ response is reliant on CNGC2.

Elongation zone epidermal cells of the *dorn1‐3* loss‐of‐function mutant, the *dorn1‐1* kinase mutant, and the *p2k2* mutant all retained a small but significant depolarization of *E*_m_ when challenged with 300 µM eATP (Fig. S4a–d; Table S2). *CNGC2* transcript levels were normal in both *dorn1‐3* and *p2k2* mutant roots, so their lowered response is most likely due to loss of receptor function rather than channel function (Fig. S4e). The *dorn1‐3p2k2* double mutant (*p2k1p2k2*) also sustained a small but significant depolarization of *E*_m_ when challenged with 300 µM eATP, but this was not significantly different to that caused by the Na_2_SO_4_ control (Fig. S5a–c; Table S2; *P* = 0.74). Under control conditions, the *p2k1p2k2* mutant had a significantly more negative *E*_m_ (−143.9 ± 4.3 mV; *n* = 10) than its paired Col‐0 wild‐type (−129.9 ± 4.6 (*n* = 5); *P* = 0.005), and this may help explain why sodium ions (Na^+^) caused a depolarization in this mutant but not in Col‐0. Overall, the results suggest that the two receptors working together are sufficient to initiate the eATP‐induced depolarization of *E*_m_ and that CNGC2 is an absolute requirement in this cell type.

Cyclic Nucleotide‐Gated Channel2 has been shown to interact with CNGC4 in immune signalling (Chin *et al*., 2013; Tian *et al*., 2019). Here, the root elongation zone epidermis of the *cngc4‐*5 loss‐of‐function mutant (Fig. S6a–d) was impaled and tested with 300 µM eATP. The eATP caused a significant depolarization of *E*_m_ to −69.4 ± 10.9 mV, similar to Col‐0 wild‐type (*P* > 0.05; Fig. S6e–g; Table S2). These results show that CNGC2 controls the PM *E*_m_ response to eATP without the need for CNGC4.

### Plasma membrane calcium‐ion currents induced by extracellular ATP in Col‐0 root epidermal protoplasts require CNGC2

**Fig. 2:**
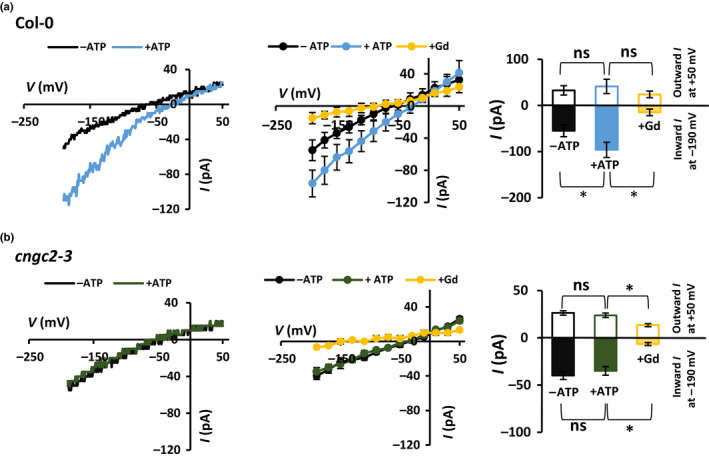
Extracellular ATP (eATP) activates inward currents in *Arabidopsis* Col‐0 but not *cngc2‐3* root elongation zone epidermal protoplasts. (a) Left panel: typical whole‐cell plasma membrane currents in Col‐0 protoplasts before (−; black) and after (+; light blue) application of 300 µM eATP. Extracellular ATP effects were observed 30 s to 3 min after addition. Bath solution contained 50 mM calcium chloride, 1 mM potassium chloride (KCl), and 10 mM MES–Tris (pH 5.6). Pipette solution comprised 5 mM barium chloride, 20 mM KCl, and 10 mM HEPES–Tris (pH 7.5). Centre panel: mean ± SE current‐voltage (*I*–*V*) relationships of Col‐0 before (−), after (+) ATP and in 100 µM gadolinium ions (Gd^3+^; dark blue; the calcium channel blocker was applied after eATP treatment) (*n* = 4). Right panel: comparison of the inward currents at −190 mV (solid bars) and the outward currents at +50 mV (hollow bars) before and after eATP addition and in the presence of Gd^3+^. Gd^3+^ block of control inward currents is also evident. (b) As (a), but for *cngc2‐3* protoplasts. The mutant did not respond to eATP even with an extended observation period (10 min). Data are means ± SE (*n* = 4; *, *P* < 0.05; ns, not significant).

Whole‐cell currents across the PM of root elongation zone epidermal protoplasts Wang *et al*. (2019) of Col‐0 and *cngc2‐3* were recorded. No significant differences in control currents or reversal potential were found between genotypes (mean ± SE reversal potential: Col‐0 −59 ± 16.3 mV, *n* = 4; *cngc2‐3* −35 ± 8.9, *n* = 4). For Col‐0, 300 µM eATP activated whole‐cell inward current upon membrane hyperpolarization, but not outward current upon membrane depolarization (Fig. 2a). No effect of Na^+^ as the salt control was found in previous trials (Wang *et al*., 2018, 2019). Analysis of the reversal potential of eATP‐activated currents (average control (no ATP) currents were subtracted from average eATP‐activated currents (Wang *et al*., 2013)) revealed an approximate value of +22 mV (*n* = 4), far from the equilibrium potentials of potassium ions (K^+^; −79 mV) and chloride ions (−28 mV) and indicating Ca^2+^ permeability. Extracellular ATP‐activated inward current was significantly inhibited by 100 µM gadolinium ions (Gd^3+^), a plant Ca^2+^ channel blocker that is effective against CNGC2 (Demidchik *et al*., 2009; Wang *et al*., 2018, 2019; Tian *et al*., 2019; Fig. 2a). These results suggest that Ca^2+^ influx across the PM contributed to the eATP‐activated current in Col‐0. As Gd^3+^ is an effective blocker of a variety of PM Ca^2+^‐permeable channels (Demidchik *et al*., 2002, 2009; Wang *et al*., 2018, 2019) it is likely that it also blocked Ca^2+^‐permeable channels that were *not* activated by eATP, causing the significant reduction in inward current in the presence of both eATP and Gd^3+^ to below the control value. The eATP‐activated Ca^2+^ inward current was absent from *dorn1‐3* PM (Fig. S7). At resting *E*_m_ of Col‐0 epidermal cells (*c*. −120 mV) the eATP‐activated current would deliver Ca^2+^ to the cytosol, which would both elevate [Ca^2+^]_cyt_ and initiate depolarization. It can be inferred that some eATP‐activated Ca^2+^ influx should have occurred in membrane voltage trials at the less negative *E*_m_ caused by EGTA (−85.2 ± −5.4 mV; Figs 1(g,h), S1c) but this was not observed, further supporting the role of Ca^2+^ influx in eATP‐induced depolarization of *E*_m_. In contrast to Col‐0, PM whole‐cell currents of *cngc2‐3* (either inward or outward) failed to respond to 300 µM eATP (Fig. 2b). Gd^3+^ (100 µM) blocked inward and outward currents in the presence of eATP, but these currents were not investigated further (Fig. 2b). Thus, the results strongly suggest that the eATP‐activated inward current in Col‐0 would be due to the hyperpolarization‐activated Ca^2+^ influx through CNGC2, helping to explain how eATP failed to depolarize the *E*_m_ of the *cngc2* mutants.

### Extracellular‐ATP‐induced cytosolic free calcium ion increase in roots is impaired in *dnd1*

**Fig. 3:**
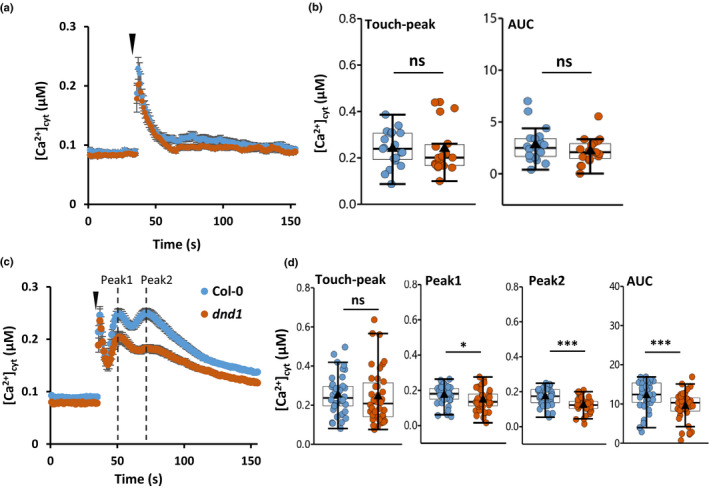
CYCLIC NUCLEOTIDE‐GATED CHANNEL2 (CNGC2) contributes to the extracellular ATP (eATP)‐induced cytosolic free calcium ion ([Ca^2+^]_cyt_) increase in Arabidopsis roots. (a) Mean ± SE [Ca^2+^]_cyt_ time‐course in control experiments (*n* = 18–19 roots in three independent trials). Sodium chloride was applied at 35 s to individual excised roots of Col‐0 or *defence not death1* (*dnd1*) (black inverted triangle; 0.6 mM final concentration). Assay solution contained 2 mM Ca^2+^ to match plasma membrane potential *E*_m_ recordings. (b) Left panel: amplitude of touch‐induced peak [Ca^2+^]_cyt_ increase after baseline subtraction. The *dnd1* response was not normally distributed, and the Mann–Whitney test was used in significance testing. Right panel: area under the curve (AUC) after baseline subtraction was analysed as an estimate of total [Ca^2+^]_cyt_ mobilized (Matthus *et al*., 2019b). (c) Mean ± SE [Ca^2+^]_cyt_ time‐course with 300 µM eATP applied at 35 s (*n* = 38 for both Col‐0 and *dnd1* in three independent trials). Dotted lines indicate time of peak response of Col‐0. (d) *dnd1* had a significantly smaller [Ca^2+^]_cyt_ response when compared with Col‐0, but not in the touch peak. The *dnd1* response for the touch peak was not normally distributed, and the Mann–Whitney test was used in significance testing. Peaks were compared with Col‐0 at the equivalent time point. Each dot in the box plots represents an individual recording. The middle line and the triangle in the box plot are the median and mean, respectively. The box outline (hinges) denotes median of the upper and the lower half of the data. The bars denote entirety of data excluding outliers; outliers are depicted by individual points outside the boxplot bars. *, *P* < 0.05; ***, *P* < 0.001; ns, not significant.

The requirement for CNGC2 in eATP‐activated epidermal PM depolarization and Ca^2+^ influx conductance should manifest in impaired eATP‐induced [Ca^2+^]_cyt_ elevation in the *dnd1* mutant, which expresses cytosolic (apo)aequorin as a bioluminescent [Ca^2+^]_cyt_ reporter. The typical monophasic [Ca^2+^]_cyt_ increase (‘touch response’) after sodium chloride (NaCl) addition (control for mechanostimulation and cation effect of Na_2_ATP) was observed in individual roots of Col‐0 and *dnd1*. The amplitude of the touch peak and total [Ca^2+^]_cyt_ mobilized did not differ significantly between genotypes (Fig. 3a). By contrast, 300 µM eATP caused a biphasic [Ca^2+^]_cyt_ increase (after the touch response) in both Col‐0 and *dnd1* roots (Fig. 3b), confirming that this part of the [Ca^2+^]_cyt_ signature was caused by eATP. This biphasic signature (‘peak 1’ and ‘peak 2’) was observed in previous studies on Arabidopsis roots and seedlings using aequorin (Demidchik *et al*., 2003; Tanaka *et al*., 2010; Matthus *et al*., 2019a,b; Mohammad‐Sidik *et al*., 2021) and also root tips using YC3.6 (Tanaka *et al*., 2010). *dnd1* roots were significantly impaired in the amplitude of both of the eATP‐induced [Ca^2+^]_cyt_ peaks and also total [Ca^2+^]_cyt_ mobilized (Fig. 3d). Significant impairment was also observed at 100 µM and 1 mM eATP (Fig. S8). Since P2K1/DORN1 governs the eATP‐induced [Ca^2+^]_cyt_ signature in Arabidopsis roots (Matthus *et al*., 2019a), impairment of the [Ca^2+^]_cyt_ response in *dnd1* helps place CNGC2 downstream of that eATP receptor, consistent with the electrophysiological data presented here.

### Root cortical plasma membrane depolarization does not require CNGC2 but may require CNGC4

The residual eATP‐induced [Ca^2+^]_cyt_ increase seen in *dnd1* roots suggests CNGC2‐independent Ca^2+^ influx pathways in other cells, such as the cortex. Cortical cells also increase [Ca^2+^]_cyt_ in response to eATP (Krogman *et al*., 2020). Cyclic Nucleotide‐Gated Channel2 redundancy was investigated by measuring elongation zone cortical cell *E*_m_. Resting Col‐0 cortical cell *E*_m_ was −131.6 ± 9.1 mV (Fig. S9a; Table S2), which was not significantly different to the epidermis. Application of eATP (300 µM) to the root transiently and significantly depolarized the cortical PM (Fig. S9a; Table S2). There was no significant difference between cortex and epidermis in terms of the maximum depolarization amplitude, the time to reach the maximum depolarization, or recovery time. The *E*_m_ of elongation zone cortical cells in the two CNGC2 mutants was then investigated. Unlike the null response of epidermal cells of *cngc2‐3* and *dnd1*, addition of eATP to the root triggered cortical *E*_m_ depolarization in both mutants (Fig. S9b,c; Table S2). No significant difference in the PM *E*_m_ before (no ATP added) or after ATP (ATP added) was observed between Col‐0 and these two mutants (Fig. S9e), indicating that CNGC2 is not involved in this cell type. The *cngc4‐5* mutant still supported a significant depolarization of cortical *E*_m_ when eATP was added to the root (Fig. S9d,e; Table S2), but this was significantly smaller than that found previously in its epidermal cells (cortex, 21.6 ± 6.8 mV; epidermis, 62.2 ± 8.8 mV; *P* = 0.012). This indicates a CNGC4‐dependent pathway in the cortex. The residual depolarization in the *cngc4‐5* implies involvement of other CNGCs (but not CNGC2) or other transport systems (Fig. S9f). Together, the results help explain the residual eATP‐induced [Ca^2+^]_cyt_ increase in *dnd1* roots; CNGC2 does not operate in all other cells.

### CNGC2 is implicated in extracellular‐ATP‐responsive gene expression

**Fig. 4:**
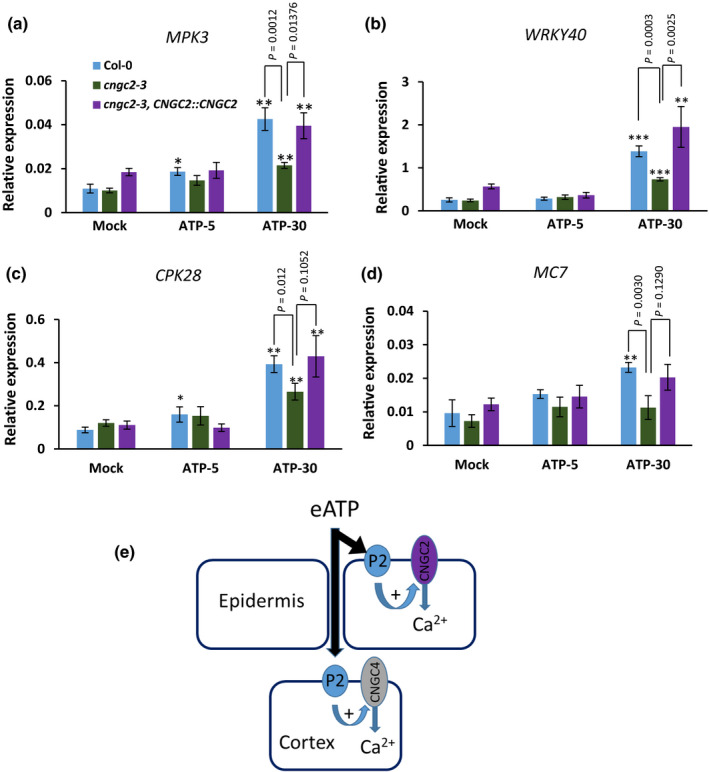
CYCLIC NUCLEOTIDE‐GATED CHANNEL2 (CNGC2) is implicated in the extracellular ATP (eATP)‐induced transcriptional response in Arabidopsis roots. Col‐0, *cngc2‐3*, and *cngc2‐3,CNGC2::CNGC2* whole roots were treated with control (sodium chloride) buffer (Mock) or 300 µM eATP for 5 min (ATP‐5) or 30 min (ATP‐30). Two housekeeping genes, *AtUBQ*10 and *AtTUB4*, were used for data normalization. Data are mean ± SE from three independent trials with *n* > 4 biological replicates. (a) Results for *MITOGEN‐ACTIVATED PROTEIN KINASE 3* (*MPK3*). (b) Results for *WRKY DNA‐BINDING PROTEIN 40* (*WRKY40*). (c) Results for *CALCIUM‐DEPENDENT PROTEIN KINASE 28* (*CPK28*). (d) Results for *METACASPASE 7* (*MC7*). Significant differences between *cngc2‐3* and the other two genotypes were found at ATP‐30, and *P* values are shown. No significant differences were observed between Col‐0 and *cngc2‐3,CNGC2::CNGC2* at ATP‐30. Asterisks indicate the statistical significance relative to the mock treatment (*, *P* < 0.05; **, *P* < 0.01; ***, *P* < 0.001). (e) Summary of possible signalling events at epidermis and cortex. DOES NOT RESPOND TO NUCLEOTIDES1 (DORN1/P2K1) and P2K2 (P2) together promote CNGC2 channel opening to mediate calcium ion (Ca^2+^) influx, plasma membrane potential *E*_m_ depolarization, and cytosolic free Ca^2+^ ([Ca^2+^]_cyt_) increase. The mechanism is unknown, but it could include phosphorylation or direct production of cyclic nucleotide monophosphates by cryptic catalytic centres (Al‐Younis *et al*., 2021). Extracellular ATP could follow the apoplastic pathway to initiate events in cortical cells, potentially through the P2 receptor complex and with CNGC4 as a component of Ca^2+^ influx, *E*_m_ depolarization and [Ca^2+^]_cyt_ increase. Other stimuli could be transmitted from the epidermis to the cortex in a CNGC2‐independent pathway.

The eATP‐responsive transcriptome is highly enriched in defence‐related and wound‐response genes, including *MITOGEN‐ACTIVATED PROTEIN KINASE 3* (*MPK3*), *WRKY DNA‐BINDING PROTEIN 40* (*WRKY40*), *CALCIUM‐DEPENDENT PROTEIN KINASE 28* (*CPK28*), and the cysteine protease *METACASPASE 7* (*MC7*) (Choi *et al*., 2014; Jewell *et al*., 2019). Transcriptional upregulation of those genes by eATP is P2K1/DORN1 dependent (Choi *et al*., 2014; Jewell *et al*., 2019), and their response to eATP was examined here in Col‐0, *cngc2‐3*, and *cngc2‐3,CNGC2*::*CNGC2* roots by qRT‐PCR. Extracellular ATP (300 µM for 30 min) significantly upregulated expression of all four genes in Col‐0, with no significant difference between Col‐0 and *cngc2‐3,CNGC2*::*CNGC2* (Fig. 4). However, transcript levels of *MPK3*, *WRKY40*, *CPK28*, and *MC7* were all significantly lower in *cngc2‐3* compared with Col‐0 or (with the exceptions of *CPK28* and *MC7*) compared with *cngc2‐3,CNGC2*::*CNGC2* (Fig. 4). Thus, CNGC2 can be required for the eATP transcriptional response.

## Discussion

Effects of eATP on plants were reported almost half a century ago (Jaffe, 1973), yet relatively few components of eATP signalling pathways have been identified. A forward genetic screen based on eATP's ability to increase [Ca^2+^]_cyt_ led to the identification of the first angiosperm eATP receptor, P2K1/DORN1 (Choi *et al*., 2014). Here, eATP's ability to depolarize root PM *E*_m_ (Lew & Dearnaley, 2000) was used in a targeted gene approach. Depolarization can arise from Ca^2+^ influx across the PM (Dindas *et al*., 2018), and eATP causes a rapid [Ca^2+^]_cyt_ increase in roots that could initiate depolarization (Waadt *et al*., 2020) as a multiconductance process (Wang *et al*., 2019). Here, eATP‐induced depolarization required extracellular Ca^2+^ (Figs 1g,h, S1c,d), showing its reliance on Ca^2+^ influx. Thus, the unresponsiveness of *cngc2* mutant root elongation zone epidermal PM to eATP (Fig. 1) is consistent with its lack of eATP‐induced PM Ca^2+^ influx currents (Fig. 2) and reveals CNGC2 as a necessary component for initiating depolarization downstream of P2K1/DORN1/P2K2 in young epidermal root cells (Fig. 4e).

Cyclic Nucleotide‐Gated Channel2 works together with CNGC4 in PAMP signalling, acting as a heterotrimeric Ca^2+^ channel in the flagellin 22 pathway (Chin *et al*., 2013; Tian *et al*., 2019). During the course of this study, Wu *et al*. (2021) reported that Arabidopsis pollen grain PM has an eATP‐activated Ca^2+^ influx conductance, measured using whole‐cell patch clamp electrophysiology. This conductance was impaired in both a single mutant of CNGC2 and a single mutant of CNGC4, suggesting that these two channel subunits might work together to facilitate germination. Whether CNGC2 and CNGC4 underpin eATP‐induced [Ca^2+^]_cyt_ elevation and transcription in pollen remains untested. Here, with eATP as a potential DAMP, CNGC2 could be acting either as a homotetramer or a heterotetramer (that includes CNGC4) in the root epidermis, but in either event it is the obligate component of the depolarization response given CNGC4's redundancy (Fig. S5e–g; Table S2). If a heterotetramer included CNGC4 (which is expressed at almost half the level of CNGC2 in the epidermis; Dinneny *et al*., 2008), that CNGC4 subunit could be replaced. This is in contrast to CNGC4's pivotal role in the PAMP signalling CNGC2/4 heterotetramer, where CNGC4 is the phosphorylation target of the BIK1 kinase (Tian *et al*., 2019).

A residual [Ca^2+^]_cyt_ signature and a transcriptional response were still observed in CNGC2 mutants, showing that other channels are involved in the root's overall response to eATP that now need to be identified. The results here from the cortex implicate a role for CNGC4 (Figs 4e, S9f). Annexin1 is implicated at whole root level, but its mode of action is not yet determined (Mohammad‐Sidik *et al*., 2021). Extracellular ATP's upregulation of defence‐related and wound‐response genes *MPK3*, *WRKY40*, *CPK28*, and *MC7* is P2K1/DORN1 dependent (Choi *et al*., 2014; Jewell *et al*., 2019) and was significantly impaired here in *cngc2‐3* (Fig. 4). Metacaspase 7 expression can be upregulated by the necrotrophic fungus *Alternaria brassicicola* (Kwon & Hwang, 2013). Its CNGC2‐dependent upregulation by eATP may relate specifically to DAMP signalling following ATP release by damaged cells. Wounded root cells not only release ATP (Dark *et al*., 2011) that could act as a DAMP for their neighbours but also release another DAMP, the peptide PLANT ELICITOR PEPTIDE 1 (PEP1; Hander *et al*., 2019). This is perceived in neighbouring cells by the cognate PM receptors PEP1 RECEPTOR 1 (PEPR1) and PEPR2 that relay to CNGC2 to cause [Ca^2+^]_cyt_ elevation (Qi *et al*., 2010). *PEPR2* is coexpressed with *P2K1/DORN1* (Tripathi *et al*., 2017). Extracellular ATP also upregulates *PEPR1* and *PEPR2* transcription (Jewell *et al*., 2019), so CNGC2 could be a common component in these DAMP pathways to facilitate the adaptive response.

## Author contributions

JS, BM, NL‐F, VL, GS, JMD: Project conception. LW, YN, JS, REM, EM, KAW, AD, A‐AV, LR, KY, WM, JMD: Experimental design, execution, and analyses. All authors contributed to writing.

## Supporting information


**Fig. S1** Controls for depolarization of elongation zone epidermis and effect of extracellular Ca^2+^ chelation or channel block.
**Fig. S2** Growth of *cngc2‐3* and receptor expression.
**Fig. S3** Extracellular ATP (eATP) did not depolarize *dnd1* elongation zone epidermis.
**Fig. S4** Single receptor mutants supported a small but significant extracellular ATP (eATP)‐induced depolarization of elongation zone epidermal *E*_m_.
**Fig. S5** The *p2k1p2k2* double receptor mutant lacked the extracellular ATP (eATP)‐induced depolarization of elongation zone epidermal *E*_m_.
**Fig. S6** *cngc4‐5* supported a significant extracellular ATP (eATP)‐induced depolarization of elongation zone epidermal *E*_m_.
**Fig. S7** Extracellular ATP (eATP) did not activate inward currents in *dorn1‐3* root elongation zone epidermal protoplasts.
**Fig. S8** Cyclic Nucleotide‐Gated Channel2 (CNGC2) contributed to the extracellular ATP (eATP)‐induced [Ca^2+^]_cyt_ increase in roots.
**Fig. S9** Cyclic Nucleotide‐Gated Channel2 (CNGC2) is not required for extracellular ATP (eATP)‐induced depolarization of primary root elongation zone cortical plasma membrane potential but CNGC4 is involved.
**Methods S1** Genotyping *cngc* insertional and complemented mutants.
**Methods S2** Growth conditions.
**Methods S3** Membrane voltage measurement.
**Methods S4** Patch clamp recordings.
**Methods S5** Quantitative real‐time PCR analysis of gene expression.
**Table S1** Primers used for genotyping transfer DNA mutant lines and quantitative real‐time PCR.
**Table S2** Mean ± SE membrane voltage *E*_m_ measurements.
Please note: Wiley Blackwell are not responsible for the content or functionality of any Supporting Information supplied by the authors. Any queries (other than missing material) should be directed to the *New Phytologist* Central Office.

## Data Availability

All lines and data will be made available in a timely manner upon request.
