# Supplementary material for: Arabidopsis thaliana CYCLIC NUCLEOTIDE‐GATED CHANNEL2 mediates extracellular ATP signal transduction in root epidermis
Source: New Phytol. 2022 Feb 20;234(2):412–21. doi: 10.1111/nph.17987 (PMC9211375; doi:10.1111/nph.17987)
Supplement: Supplementary file 1 — Fig. S1 Controls for depolarization of elongation zone epidermis and effect of extracellular Ca2+ chelation or channel block. Fig. S2 Growth of cngc2‐3 and receptor expression. Fig. S3 Extracellular ATP (eATP) did not depolarize dnd1 elongation zone epidermis. Fig. S4 Single receptor mutants supported a small but significant extracellular ATP (eATP)‐induced depolarization of elongation zone epidermal E m. Fig. S5 The p2k1p2k2 double receptor mutant lacked the extracellular ATP (eATP)‐induced depolarization of elongation zone epidermal E m. Fig. S6 cngc4‐5 supported a significant extracellular ATP (eATP)‐induced depolarization of elongation zone epidermal E m. Fig. S7 Extracellular ATP (eATP) did not activate inward currents in dorn1‐3 root elongation zone epidermal protoplasts. Fig. S8 Cyclic Nucleotide‐Gated Channel2 (CNGC2) contributed to the extracellular ATP (eATP)‐induced [Ca2+]cyt increase in roots. Fig. S9 Cyclic Nucleotide‐Gated Channel2 (CNGC2) is not required for extracellular ATP (eATP)‐induced depolarization of primary root elongation zone cortical plasma membrane potential but CNGC4 is involved. Methods S1 Genotyping cngc insertional and complemented mutants. Methods S2 Growth conditions. Methods S3 Membrane voltage measurement. Methods S4 Patch clamp recordings. Methods S5 Quantitative real‐time PCR analysis of gene expression. Table S1 Primers used for genotyping transfer DNA mutant lines and quantitative real‐time PCR. Table S2 Mean ± SE membrane voltage E m measurements. Please note: Wiley Blackwell are not responsible for the content or functionality of any Supporting Information supplied by the authors. Any queries (other than missing material) should be directed to the New Phytologist Central Office. [file NPH-234-412-s001.pdf]

New Phytologist

New Phytologist **Supporting Information**

Article title: *Arabidopsis thaliana* Cyclic Nucleotide-Gated Channel2 mediates extracellular ATP signal transduction in root epidermis.
Authors: Limin Wang, Youzheng Ning, Jian Sun, Katie A. Wilkins, Elsa Matthus, Rose E. McNelly, Adeeba Dark, Lourdes Rubio, Wolfgang Moeder, Keiko Yoshioka, Anne-Aliénor Véry, Gary Stacey, Nathalie Leblanc-Fournier, Valérie Legué, Bruno Moulia, Julia M. Davies.
Article acceptance date: 16 January 2022

The following Supporting Information is available for this article:

Methods S1 Genotyping *cngc* insertional and complemented mutants.

Arabidopsis genomic DNA was isolated from 14-day-old plants. After freezing in liquid nitrogen, samples were ground in buffer containing 1M Tris (pH adjusted to 7.5 with HCl); 1M NaCl; 0.5M EDTA (pH adjusted to 8.0 with NaOH); 10 % (w/v) SDS. PCR was performed using the primer pairs listed in Table S1. PCR conditions were as follows: 35 cycles at 94°C for 30 s, 61°C for 30 s, and 72°C for 2 min. The amplified gene products were then visualised by gel electrophoresis (0.8 % (w/v) agarose in 1X TAE buffer). The gel was stained with SYBR Safe DNA Gel Stain (Invitrogen). The *cngc2-3* complementation lines were generated using the CNGC2 cDNA fragment that was fused to the 1.5 kb endogenous promoter sequence including 5'UTR and intron 1 in pORE-01. The complementation lines were referred to as *cngc2-3,CNGC2::CNGC2*. Genotyping was conducted by the primer combination listed in Table S1 (722 bp amplicon for the transgene, 1076 bp amplicon for the endogenous gene).

Table S1 Primers used for genotyping T-DNA mutant lines and qRT-PCR.

Gene	Forward (5'-3')	Reverse (3'-5')
**Genotype**		
cngc2-3	GCGTGGACCGCTTGCTGCAACT (LBb1-F)	CTGCAGCCTTAGACCAAGCCCGACC
cngc2-3, CNGC2::CNGC2	CCAGCCCGAACCAGATTCCGACGTCT	GGCCCTTGACGTTAGACTCAGACCG
CNGC4	CTGTTGTGCTCTCCAAATTCC	GTTACCTTTTCCGGGTACACT
cngc4-5	GTTCCGAAATCGGCAAAAT (LB1.3)	GTTACCTTTTCCGGGTACACT
**qPCR**		
P2K1/DORN1**(AT5G60300)**	TGGAGTTTGTCAGGTCCATCG	AACGGACGTCTTCTAGGAGTC
P2K2 **(AT3G45430)**	GGTTTCATGACCATGGAGGCA	CACCTTAAGCCCCACGTTCA
WRKY40 **(AT1G80840)**	AGCTTCTGACACTACCCTCGTTG	TTGACAGAACAGCTTGGAGCAC

CNGC2 **(AT5G15410)**	TCTTCAGGTGGATTGGACTGT	TCCACCGTTGATTTGGAGGT
MPK3 **(AT3G45640)**	TGGAGCTTATGGAATCGTTTGCTC	TCATCGCTACTAGCTCGTTCGTC
CPK28 **(AT5G66210)**	GCCTGAGGAACTTCGAATGCAC	TGTCTGCTTCATCCAGCAGTGG
MC7 **(AT1G79310.1)**	GGAAACAGGGGAAGAGGATG	CCGCTGTGACAAGAGTCTGA
UBQ10 **(AT4G05320)**	CCGACTACAACATTCAGAAGGA	TCAGAACTCTCCACCTCCAAA
TUB4 **(AT5G44340)**	AGGGAAACGAAGACAGCAAG	GCTCGCTAATCCTACCTTTGG

Methods S2 Growth conditions.

Surface-sterilised seeds were grown on one-half strength (½) Murashige-Skoog (MS) medium including vitamins (Duchefa), 1 % (w/v) sucrose, 0.7 or 0.8 % (w/v) Bacto-agar, with pH adjusted to 5.6 or 5.7 with KOH. After 2 days of stratification in darkness at 4 °C, they were transferred into a growth chamber (16 h light/ 8 h dark, 21 to 23℃) with light intensity of 80 to 140 µmol $m^{-2} \cdot s^{-1}$. For biomass assays, seeds were transferred to soil after 14 days and grown under long-day conditions (16 h light/ 8 h dark) in a growth room (200 µmol m^{-2} s^{-1} light intensity, 20 °C, 60 % relative humidity).

Methods S3 Membrane voltage measurement.

The method was adapted from Demidchik *et al*. (2002) and Drain *et al*. (2020). Microelectrodes were fabricated from borosilicate capillaries (Harvard, G200F-4 or GC200F-10) with a vertical puller (PC-10 Narishige, East Meadow, NY) and backfilled with 1M KCl. Microelectrodes were connected via an Ag/AgCl pellet to the probe of an electrometer (WPI773, Word Precision Instruments, Sarasota, FL or Axoprobe 1A, Axon Instruments, Foster City, USA). The reference comprised a capillary tube containing 1 M KCl in 2 % (w/v) agar, connected into an Ag/AgCl electrode, and placed in the chamber. The microelectrode was placed at the surface of elongation zone using a manually operated micromanipulator and impalement into a root cell was achieved either by movement of the chamber using a micro-elevator (IT6D CA1 Microcontrol, Newport or with the manually operated micromanipulator. Data at three minutes before chemical application were analyzed and shown. Data were recorded at least for 9 minutes' test treatment. An individual root was only used for one impalement.

Demidchik V, Bowen HC, Maathuis FJ, Shabala SN, Tester MA, White PJ, Davies JM. 2002. *Arabidopsis thaliana* root non-selective cation channels mediate calcium uptake and are involved in growth. *Plant Journal* **32**: 799-808.

Drain A, Thouin J, Wang L, Boeglin M, Pauly N, Nieves-Cordones M, Gaillard I, Véry AA, Sentenac H. 2020. Functional characterization and physiological roles of the single Shaker outward K^+ channel in *Medicago truncatula*. *Plant Journal* **102**:1249-1265.

Methods S4 Patch clamp recordings.

Root tips (2-3 mm) were cut from Col-0, *cngc2-3* or *dorn1-3* plants. They were directly incubated in an enzyme solution containing 1 % (w/v) cellulysin (Calbiochem), 1 % (w/v) cellulase RS (Yakult Honsha) , 0.1 % (w/v) pectolyase Y-23 (Yakult Honsha), 0.1 % (w/v) bovine serum albumin (BSA), 10 mM $CaCl_2$, 10 mM KCl, 2 mM $MgCl_2$, 2 mM MES and 165 mM D-sorbitol, pH 5.6 with Tris. After 1h 40 mins' incubation at room temperature in the dark, protoplasts were filtered through a 40 µm nylon mesh and washed with holding buffer twice by slow-speed centrifugation at 4 ℃. The holding buffer comprised 0.2 mM $CaCl_2$, 0.1 mM KCl, 10 mM MES, pH 5.6 with Tris and 280 mosmol·L^{-1} with D-sorbitol. Protoplasts were suspended in this holding buffer and stored on ice (dark) until experiments. After using the N9093 epidermal-specific green fluorescent protein reporter line to establish the isolation protocol (Wang *et al*., 2019), protoplast origin was confirmed by direct observation of the genotypes listed above for testing. Apparatus was as described by Demidchik *et al.* (2002); see Methods S3 for reference. Patch-clamp experiments were performed in the whole-cell configuration. Pipette resistance was 10 MOhms. After a gigaohm seal was formed in a cell-attached configuration, the whole-cell configuration was achieved by gentle suction. Whole-cell currents were then recorded after at least 12 minutes. A ramp protocol from +50 to -190 mV (200 mV·S^{-1}) with a holding potential at -35 mV (corrected for liquid junction potential) was used to elicit currents. The bath solution contained (mM): 50 $CaCl_2$, 1 KCl, 10 MES-Tris, pH 5.6. The pipette solution comprised (mM): 5 $BaCl_2$, 20 KCl, 10 HEPES-Tris, pH 7.5. The osmolarity of bath and pipette solutions was adjusted to 280 mosmol·L^{-1} and 290 mosmol·L^{-1} respectively with D-sorbitol. Ionic strength was determined using the calculator integrated in the "Calcium.exe" program (Föhr *et al*., 1993). Thereafter, ion activities were calculated using GEOCHEM (Parker *et al*., 1994). Equilibrium potentials were calculated using the Nernst equation.

Föhr KJ, Warchol W, Gratzl M. 1993. Calculation and control of free divalent cations in solutions used for membrane fusion studies. *Methods Enzymology* **221:** 149-157.

Parker DR, Norvelll WA, Chaney RL. 1994. A chemical specition program for IBM and compatible computers. *Chemical Equilibrium and Reaction Models. Eds. RH Loepper et al. SSSA Spec Pub No Soil Science Society of America, Madison, WI.*

Methods S5 qRT-PCR analysis of gene expression.

In tests of basal expression of *CNGC2* in eATP receptor mutants (*dorn1-3* and *p2k2*) or expression of receptors (*DORN1-3* and *P2K2*) in *cngc2-3*, 25 roots of a genotype (one plate) were harvested and frozen in liquid nitrogen as one RNA extraction. Each trial had 3-4 plates per genotype. Three trials were repeated. Transcripts were detected with the primers given in Table S1. *AtUBQ*10 and *AtTUB4* were used for data normalization according to the following equation (Swarbreck *et al*., 2019):

$$R_{gene} = Efficiency^{-Ct} / \sqrt{(R_{UBQ10}) * (R_{TUB4})}$$

In tests of eATP-regulated expression, 25 plants (one plate) of 11-day-old Col-0, or *cngc2-3* or *cngc2-3,CNGC2::CNGC2* were pooled together. Roots were acclimatised in the growth chamber in a 2 mL Eppendorf tube containing ½ MS solution, pH 5.6. After 1.5 hours, the ½ MS solution was discarded by pipetting and replaced by either 600 µM NaCl control buffer or 300 µM eATP solution (prepared in ½ MS solution, pH 5.6). After 5 and 10 min, roots from each tube (as one biological repeat) were quickly dried with tissue, excised into an Eppendorf tube and frozen in liquid nitrogen. RNA extraction and qRT-PCR were performed as described above. Three trials were repeated.

Swarbreck SM, Guerringue Y, Matthus E, Jamieson FJ, Davies JM. 2019. Impairment in karrikin but not strigolactone sensing enhances root skewing in *Arabidopsis thaliana*. *Plant J.* **98:** 607-621.

Table S2 Mean ± SE membrane voltage (E_m) measurements.

Genotype	Resting E_m (mV)	E_m (+ATP; (mV))	*p* value (Student's paired *t*-test)	Time at the maximum depolarization (minutes)
**Epidermis**				
Col-0 (*#1*)	-118.9 ± 4.8 (*n*=9)	-69.2 ± 7.6 (*n*=9)	3.55E-05	1.82 ± 0.27 (*n*=9)
cngc2-3	-125.7 ± 5.8 (*n*=9)	-122.1 ± 4.7 (*n*=9)	0.079652	NA
cngc2-3,CNGC2::CNGC2	-118.2 ± 4.9 (*n*=5)	-54.7 ± 7.2 (*n*=5)	0.000425	0.90 ± 0.04 (*n*=5)
dnd1	-122.2 ± 3.2 (*n*=10)	-122.7 ± 3.2 (*n*=10)	0.653006	NA
cngc4-5	-131.6 ± 5.9 (*n*=7)	-69.4 ± 11.0 (*n*=7)	0.000395	1.29 ± 0.16 (*n*=7)
Col-0 *(#2)*	-129.9 ± 4.6 (*n*=5)	-60.6 ± 2.7 (*n*=5)	0.00029	1.54 ± 0.48 (*n*=5)
dorn1-3	-126.4 ± 5.5 (*n*=5)	-120.9 ± 4.3 (*n*=5)	0.045094	0.40 ± 0.08 (*n*=5)
dorn1-1	-129.2 ± 5.0 (*n*=5)	-107.5 ± 6.3 (*n*=5)	0.019933	1.38 ± 0.24 (*n*=5)
p2k2	-123.6 ± 4.4 (*n*=5)	-117.7 ± 4.1 (*n*=5)	0.014446	1.66 ± 0.33 (*n*=5)
p2k1p2k2 (a)	-141.8 ± 7.4 (*n*=4)	-135.2 ± 9.4 (*n*=4)	0.022708	3.78 ± 1.29 (*n*=4)
p2k1p2k2 (b)	-145.3 ± 5.7 (*n*=6)	-135.2 ± 5.8 (*n*=6)	0.001513	3.22 ± 0.32 (*n*=6)
Cortex				
Col-0 *(#1)*	-131.6 ± 9.1 (*n*=5)	-80.3 ± 6.5 (*n*=5)	0.002861	1.96 ± 0.83 (*n*=5)
cngc2-3	-122.7 ± 4.3 (*n*=4)	-62.5 ± 9.2 (*n*=4)	0.003052	2.08 ± 0.86 (*n*=4)
dnd1	-115.9 ± 5.9 (*n*=3)	-81.5 ± 11.1 (*n*=3)	0.021657	2.27 ± 0.58 (*n*=3)
cngc4-5	-123.6 ± 8.4 (*n*=4)	-102.0 ± 9.0 (*n*=4)	0.02534	2.65 ± 0.51 (*n*=4)

Note: Col-0 (#1) is the Columbia wild type paired for *CNGC* mutants; Col (#2) is the Columbia wild type paired for eATP receptors mutants. For the *p2k1p2k2* mutant, (a) is the response to 300 µM Na_2SO_4 and (b) is the response to 300 µM eATP. *p* value indicates the statistical comparison between the resting E_m (-ATP) and E_m at the maximum depolarization (+ATP) by the Student's two tailed paired *t*-test in a given genotype. For *p2k1p2k2* (a) the value is for the effect of Na_2SO_4. NA; not applicable.

Fig. S1 Controls for depolarisation of elongation zone epidermis and effect of extracellular Ca^{2+} chelation or channel block.

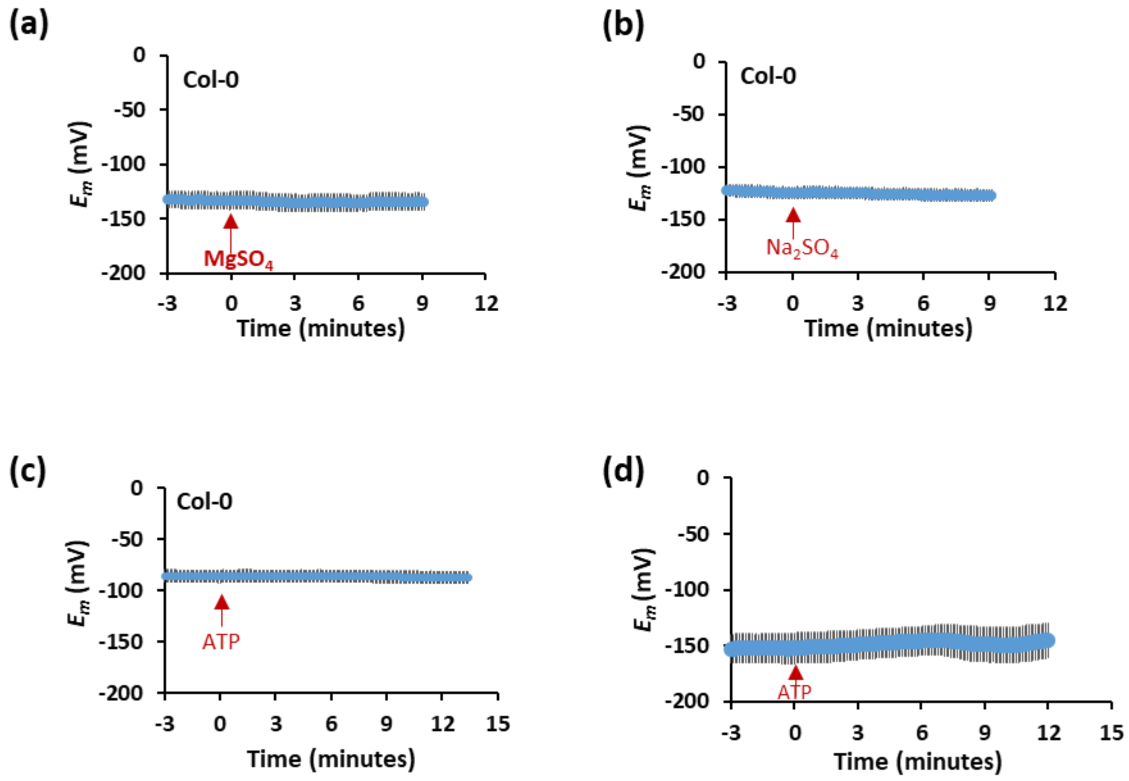

(a) Mean ± SE time course of elongation zone epidermal E_m recording from *Arabidopsis* Col-0 treated with 300 µM $MgSO_4$ as a control for Mg^{2+} addition in MgATP tests (red triangle indicates addition; $n = 8$). (b) As (a) but with 300 µM Na_2SO_4 ($n = 6$). (c) Addition of 300 µM eATP to a bathing medium containing 5 mM EGTA to chelate extracellular Ca^{2+} ($n = 5$). (d) Addition of 300 µM eATP to a bathing medium containing 0.5 mM $LaCl_3$ to block PM Ca^{2+}-permeable channels ($n = 3$).

Fig. S2 Growth of *cngc2-3* and receptor expression.

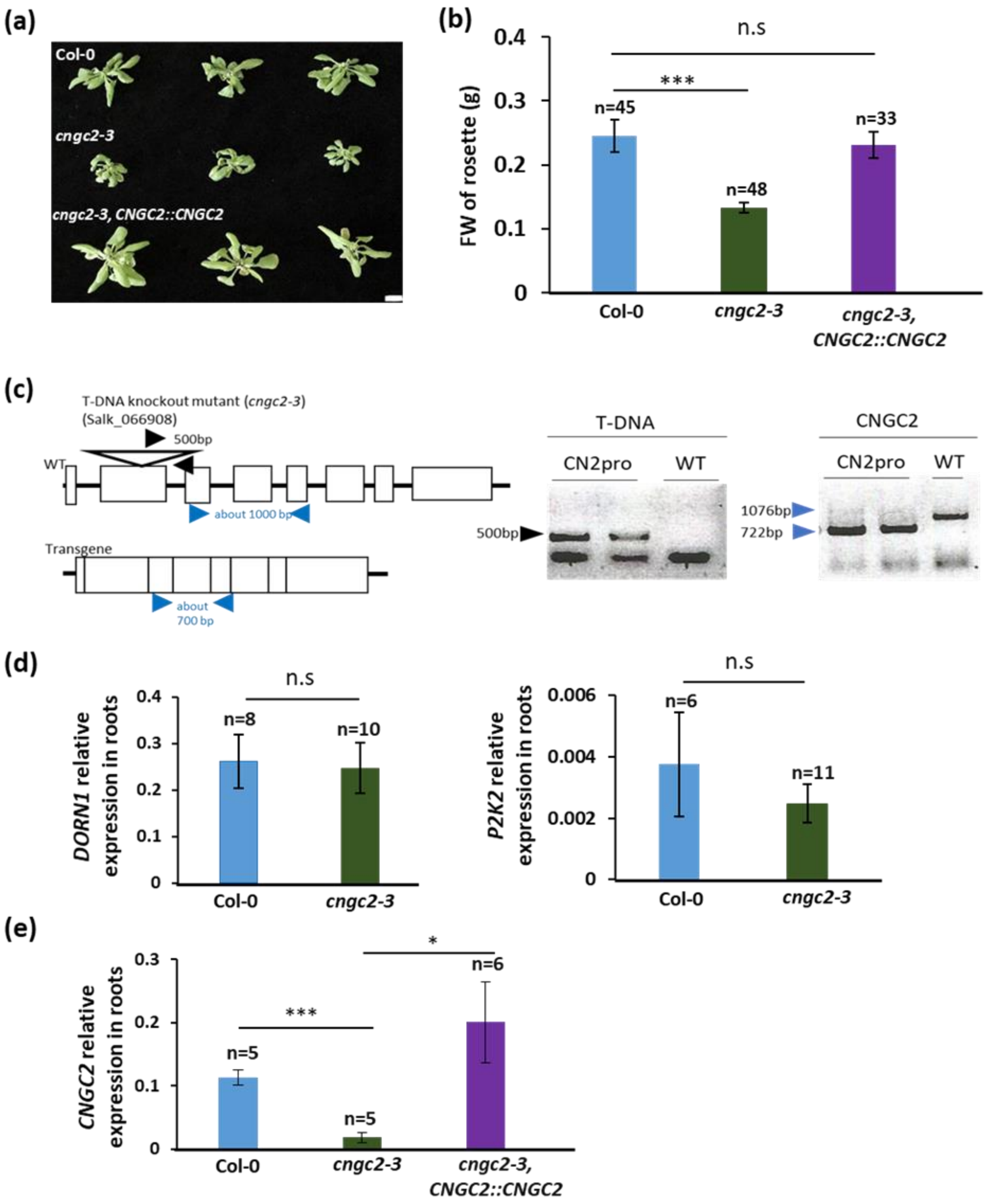

(a) Rosette morphology of 4-week-old *Arabidopsis* Col-0, *cngc2-3* and *cngc2-3,CNGC2*::*CNGC2* grown in soil at 20 °C. *cngc2-3* displayed its phenotypical dwarf growth (Chin *et al*., 2013) which was restored to normal by complementation. Scale bar = 1 cm. (b) Fresh weight of 4-week-old

plants. Numbers indicate total individual plants harvested in 3 trials. Complementation restored normal biomass to the mutant. (c) Genotyping of *cngc2-3*. The T-DNA insertion was confirmed by PCR using primers listed in Table S1. Primer positions (left), and PCR result (right) CN2pro: complemented lines. Genotyping was conducted by the primer combination listed in Table S1 (722 bp amplicon for the transgene, 1076 bp amplicon for the endogenous gene). (d) Relative *CNGC2* expression in 11-day old roots of Col-0, *cngc2-3* and *cngc2-3,CNGC2*::*CNGC2*. Numbers indicate total number of biological repeats (25 plants per plate as a repeat) in 3 trials. (e) Relative *DORN1/P2K1* and *P2K2* expression in 11-day old roots of Col-0 and *cngc2-3*. Numbers indicate total number of biological repeats in 3 trials. Asterisks indicate the significance levels (*, $p < 0.05$; ***, $p < 0.001$; n.s, no significant difference).

Chin K, DeFalco TA, Moeder W, Yoshioka K. 2013. The *Arabidopsis* cyclic nucleotide-gated ion channels AtCNGC2 and AtCNGC4 work in the same signaling pathway to regulate pathogen defense and floral transition. *Plant Physiology* **163**(2): 611-624.

Fig. S3 eATP did not depolarise *dnd1* elongation zone epidermis.

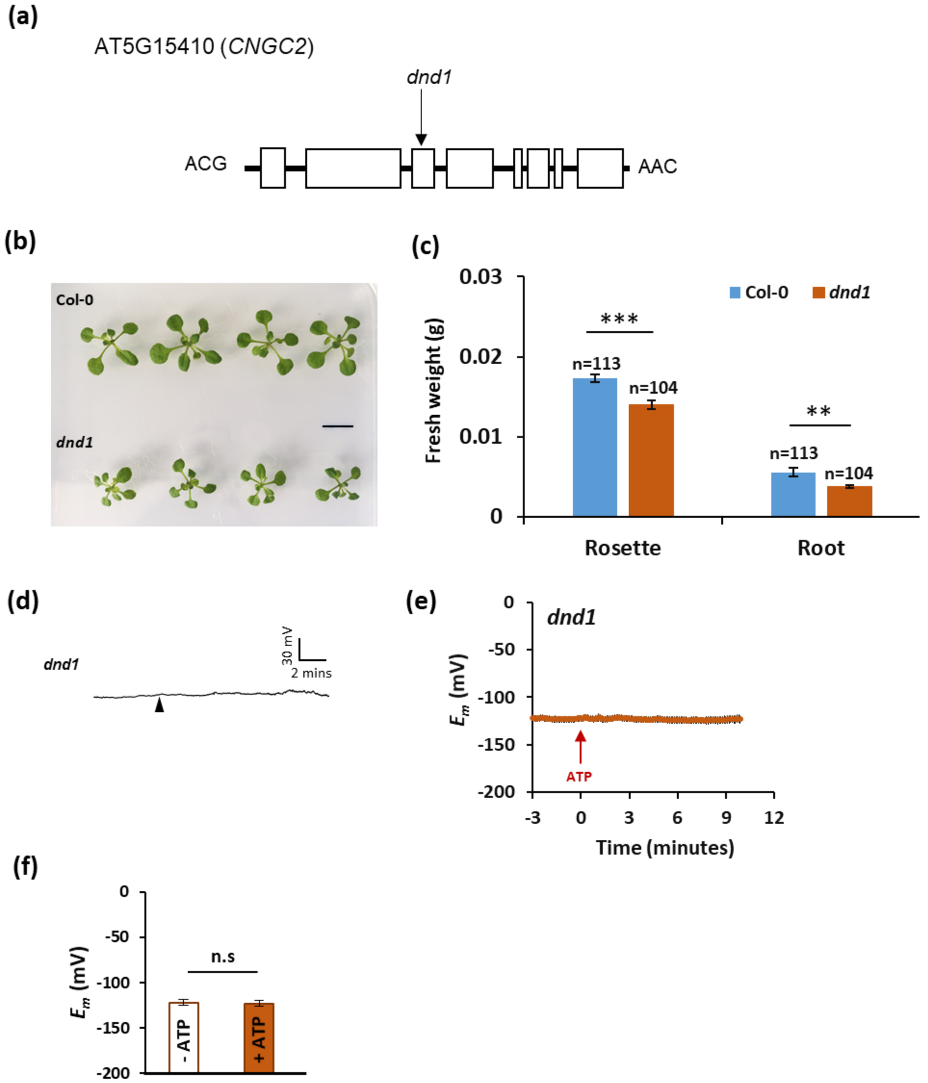

(a) The *Arabidopsis dnd1* mutant of CNGC2 has a single point mutation causing a stop codon in the third exon that would result in a truncated protein lacking the pore region for ion conduction. (b) Rosette morphology of 16-day-old Col-0 and mutant (*dnd1*) grown in ½ MS medium at 23 °C. The previously reported dwarf phenotype reported by Chin *et al*. (2013) was also observed here. (c) Fresh weight of 16-day-old plants. Numbers indicate total individual plants harvested in 3 trials. Scale bar = 1 cm. (d) Representative epidermal E_m recording from *dnd1* treated with 300 µM eATP (black triangle indicates addition). (e) Mean ± SE time course of the response to 300 µM eATP (n = 10). (f) Comparison of mean ± SE E_m before eATP (-ATP) and after eATP treatment (+ATP; maximum depolarisation). ***, $p < 0.001$; ** p, < 0.01; n.s, not significant.

Chin K, DeFalco TA, Moeder W, Yoshioka K. 2013. The *Arabidopsis* cyclic nucleotide-gated ion channels AtCNGC2 and AtCNGC4 work in the same signaling pathway to regulate pathogen defense and floral transition. *Plant Physiology* **163**(2): 611-624.

Fig. S4 Single receptor mutants supported a small but significant eATP-induced depolarisation of elongation zone epidermal E_m.

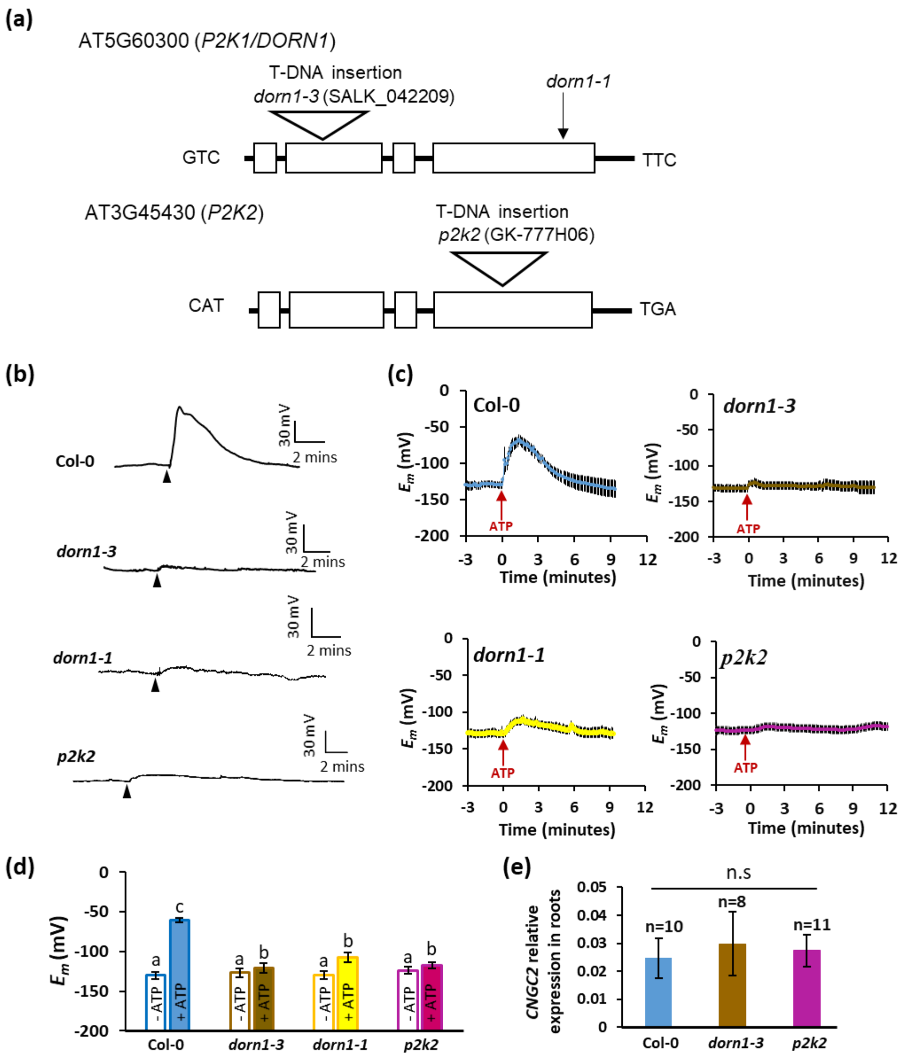

(a) The *Arabidopsis dorn1-1* loss of function mutant and *dorn1-3* kinase mutant arise from a T-DNA insert in the second exon and a single point mutation in the fourth exon respectively. The *p2k2* kinase mutant arises from a T-DNA insertion in its fourth exon. (b) Representative epidermal E_m recordings from Col-0, *dorn1-3, dorn1-1* and *p2k2* treated with 300 µM eATP (black triangles indicate addition). (c) Mean ± SE time course of the responses to 300 µM eATP. Col-0 (n = 5), *dorn1-3* (n = 5), *dorn1-1* (n = 5), *p2k2* (n = 5). (d) Comparison of mean ± SE E_m before eATP (-ATP) and after eATP treatment (+ATP; maximum depolarisation). Different letters on the top of vertical bars indicate significant difference ($p < 0.05$). (e) Relative *CNGC2* expression in 14-day old roots of Col-0, *dorn1-3* and *p2k2*. Numbers indicate total number of biological repeats in 3 trials. n.s, not significant.

Fig. S5 The *p2k1p2k2* receptor mutant lacked the eATP-induced depolarisation of elongation zone epidermal E_m.

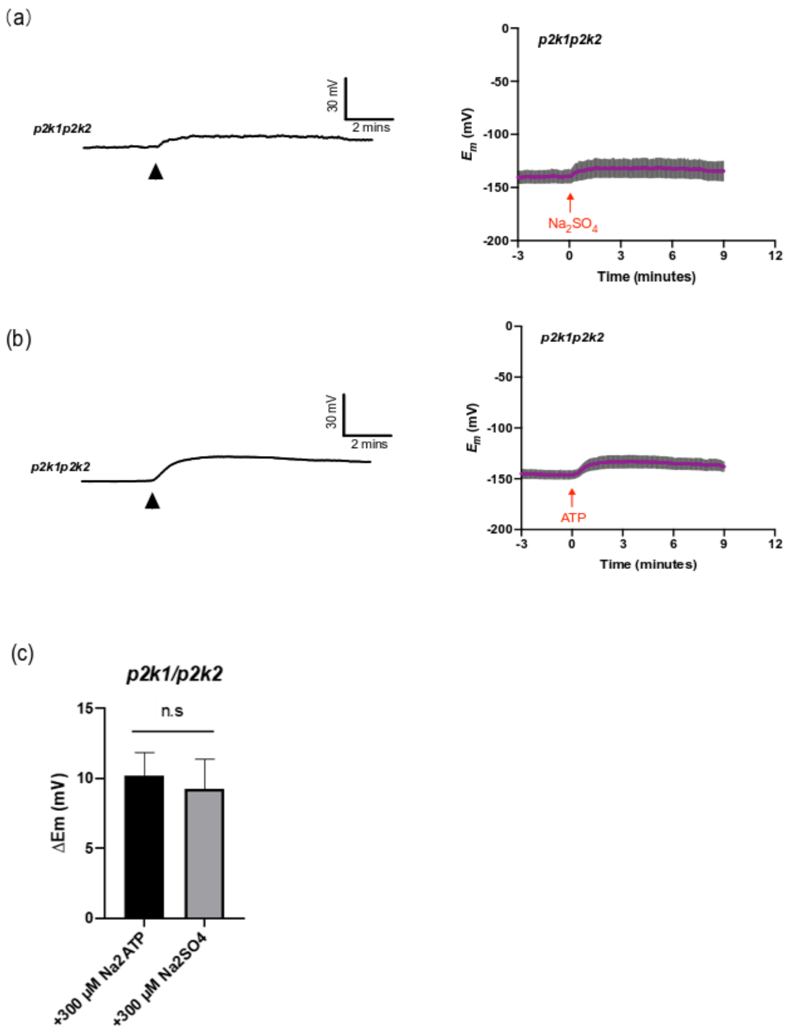

(a)Representative epidermal E_m recording and mean ± SE time course (*n* = 4) of the response from *Arabidopsis p2k1p2k2* treated with 300 µM Na_2SO_4 (black triangles indicate addition). (b) Representative epidermal E_m recording and mean ± SE time course (*n* = 6) of the response from *p2k1p2k2* treated with 300 µM eATP. (c) Comparison of mean ± SE maximum E_m depolarisations in response to 300 µM Na_2SO_4 or 300 µM eATP. n.s. indicates not significant.

Fig. S6 ***cngc4-5* supported a significant eATP-induced depolarisation of elongation zone epidermal E_m.**

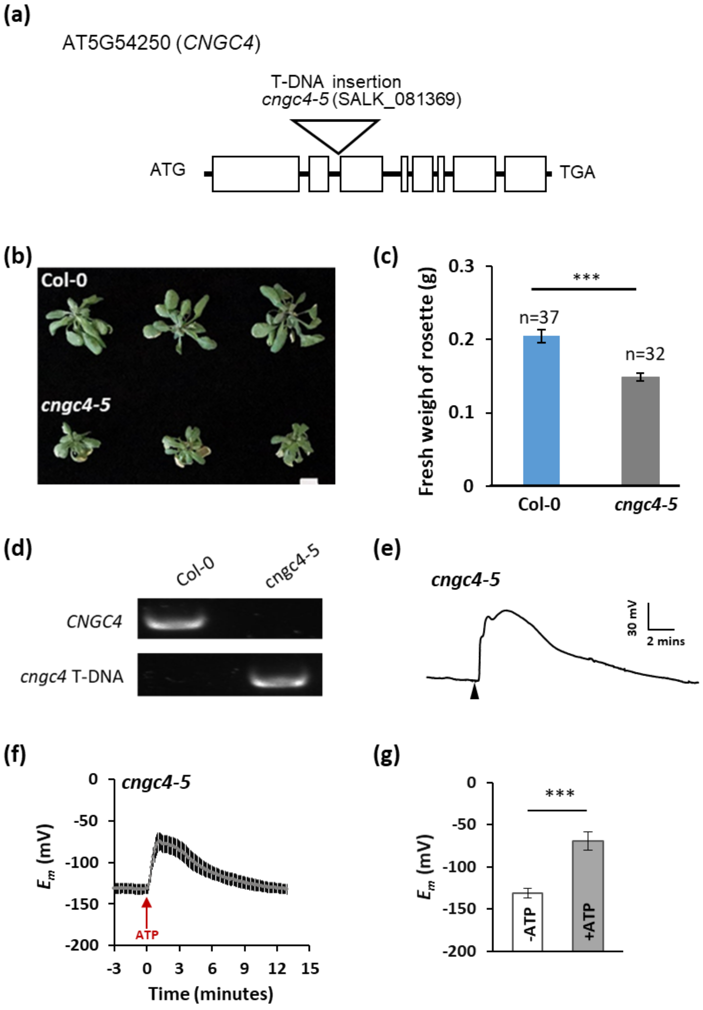

(a)The *Arabidopsis cngc4-5* mutation arises from an intron T-DNA insertion. (b) Rosette morphology of 4-weeks-old Col-0 and *cngc4-5* grown in soil at 20 °C . Scale bar = 1 cm. (c) Fresh weight of 4-week-old Col-0 and *cngc4-5* rosettes. The previously observed dwarf *cngc4-5* phenotype (Tian *et al*., 2019) was also found here. Numbers indicate total individual plants harvested in 3 trials. Asterisks indicate the significance level (***, $p < 0.001$). (d) Genotyping *cngc4-5*. Top panel*; CNGC4* transcript was absent from the *cngc4-5* mutant. Bottom panel; the T-DNA insertion in *cngc4-5* was confirmed by PCR (primers detailed in Table S1). (e) Representative elongation zone epidermal E_m recording from *cngc4-5* treated with 300 µM eATP (black triangle indicates addition). (f) Mean ± SE time course of the response to 300 µM eATP ($n = 7$). (g) Comparison of mean ± SE E_m before eATP (-ATP) and after eATP treatment (+ATP; maximum depolarisation). ***, $p < 0.001$.

Tian W, Hou C, Ren Z, Wang C, Zhao F, Dahlbeck D, Hu S, Zhang L, Niu Q, Li L, Staskowicz BJ, Luan S. 2019. A calmodulin-gated calcium channel links pathogen patterns to plant immunity. *Nature* **572**(7767): 131-135.

Fig. S7 eATP did not activate inward currents in *dorn1-3* root elongation zone epidermal protoplasts.

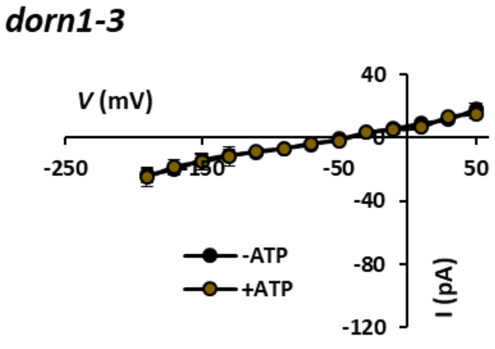

Mean ± SE current-voltage (*I*-*V*) relationships of *Arabidopsis dorn1-3* before (-), after (+) application of 300 µM eATP (*n* = 4). The bath solution contained (mM): 50 $CaCl_2$, 1 KCl, 10 MES-Tris (pH 5.6). The pipette solution comprised (mM): 5 $BaCl_2$, 20 KCl, 10 HEPES-Tris (pH 7.5). No significant differences were found between currents under control conditions between *dorn1-3*, Col-0 or *cngc2-3* and neither were there significant differences between reversal potentials. The mean ± SE reversal potential for *dorn1-3* under control conditions was -47.5 ± 2.5 mV (*n* = 4). The mutant did not respond to eATP even after a prolonged observation time (10 min).

Fig. S8 CNGC2 contributed to the eATP-induced $[Ca^{2+}]_{cyt}$ increase in roots.

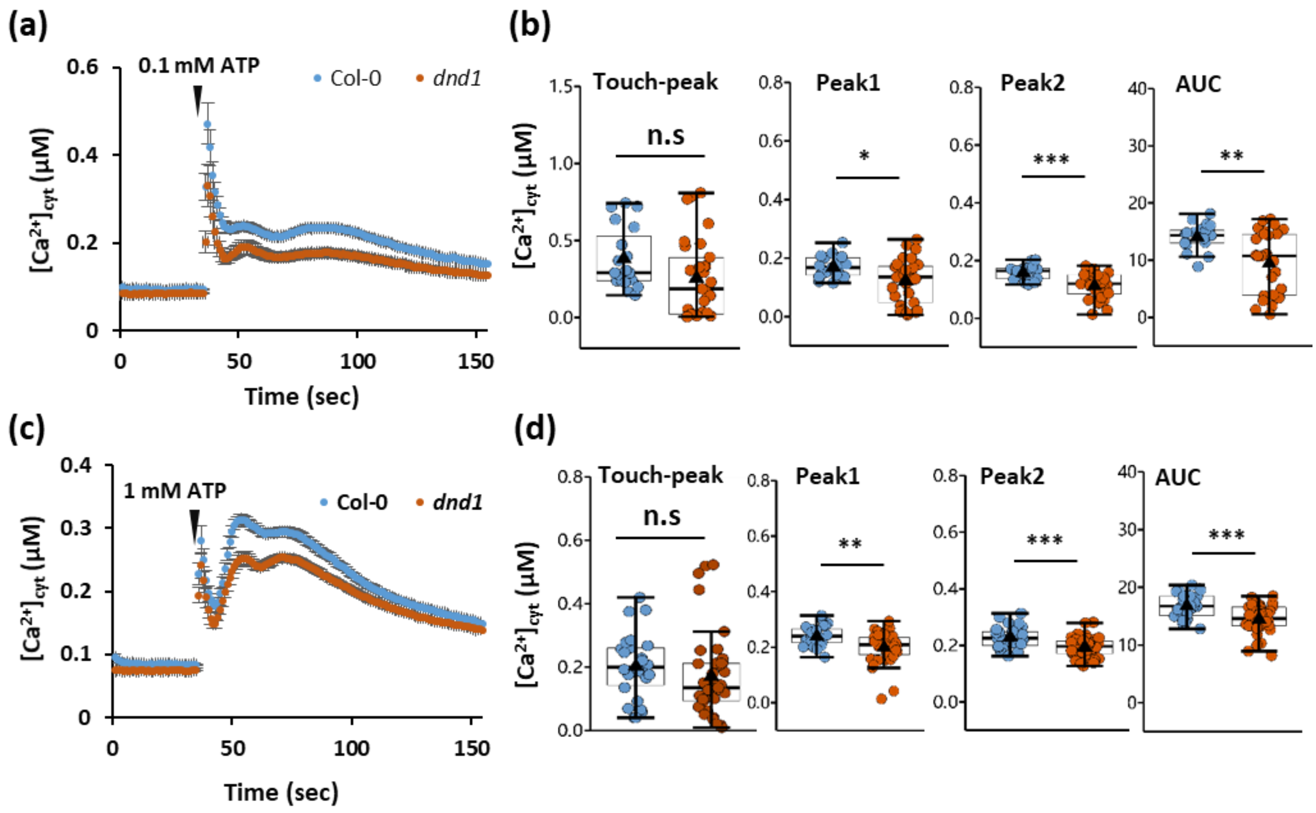

(a) Mean ± SE $[Ca^{2+}]_{cyt}$ time-course in response to 0.1 mM eATP (n = 24-35 roots in 4 independent trials). eATP was applied at 35 s to individual excised roots of *Arabidopsis* Col-0 or *dnd1* (black inverted triangle). (b) Amplitude of peak $[Ca^{2+}]_{cyt}$ increases and area under the curve (AUC) after baseline-subtraction. *dnd1* had a significantly smaller $[Ca^{2+}]_{cyt}$ response when compared to Col-0, but not in the touch-peak. (c) Mean ± SE response to 1 mM eATP applied at 35s (n = 26-39 roots in 3 independent trials). (d) *dnd1* had a significantly smaller $[Ca^{2+}]_{cyt}$ response when compared to Col-0, but not in the touch-peak. Each dot in box plots represents an individual recording. The middle line and the triangle in the box plot are the median and mean, respectively. ***, $p < 0.001$; ** p, < 0.01; *, $p < 0.05$; n.s, not significant.

Fig. S9 CNGC2 is not required for eATP-induced depolarisation of primary root elongation zone cortical plasma membrane potential but CNGC4 is involved.

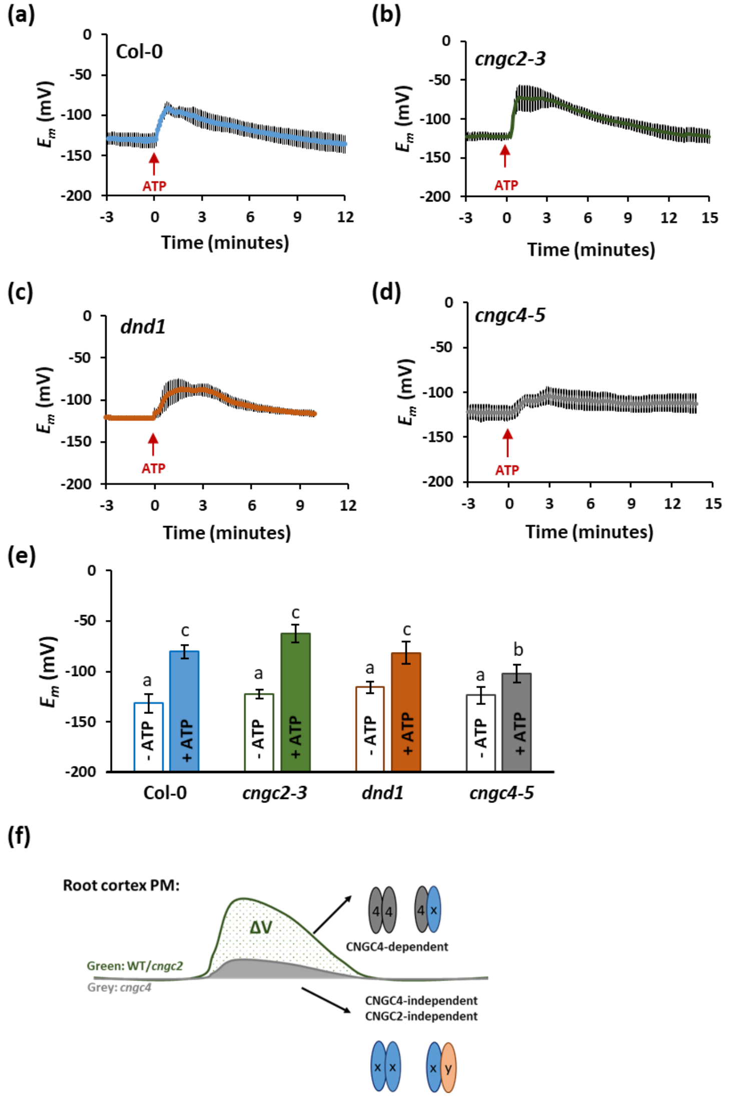

(a)eATP (300 µM) added to a root caused depolarisation of the cortical PM E_m of *Arabidopsis* Col-0 (n = 5), (b) *cngc2-3* (n = 4), (c) *dnd1* (n = 3) and (d) *cngc4-5* (n = 4). (*e*) Comparison of E_m without eATP (-ATP) and E_m at the maximum depolarisation by eATP (+ATP). Data shown are means ± SE. Different letters on the top of vertical bars indicate significant difference ($p < 0.05$). (f) CNGC4's involvement in the cortical PM's response to eATP challenge at the epidermis. Col-0 wild type (WT) and *cngc2* (green line) supported equivalent depolarisation of E_m, indicating no involvement of CNGC2 in this cell type. The smaller depolarisation in *cngc4* is shown by the grey line. The difference in depolarisation between WT/*cngc2* and *cngc4* (ΔV) must be CNGC4-dependent, with CNGC4's operating as a homomer or heteromer. For simplicity only two subunits of either possible channel complex is shown. The residual depolarisation in *cngc4* (solid grey) must be independent of both CNGC4 and CNGC2. If reliant on CNGCs, then homeric or heteromeric complexes could operate. It is also possible that other types of transporters are involved in the residual depolarisation.
